# Adipocyte-derived IL-6 and leptin promote breast Cancer metastasis via upregulation of Lysyl Hydroxylase-2 expression

**DOI:** 10.1186/s12964-018-0309-z

**Published:** 2018-12-18

**Authors:** Jin-Yong He, Xiao-Hui Wei, Si-Jing Li, Yang Liu, Hao-Lin Hu, Zheng-Zheng Li, Xin-Hong Kuang, Lai Wang, Xin Shi, Sheng-Tao Yuan, Li Sun

**Affiliations:** 1 Jiangsu Key laboratory of Drug Screening, China Pharmaceutical University, Nanjing, China; 2 Jiangsu Center for Pharmacodynamics Research and Evaluation, China Pharmaceutical University, Nanjing, China; 3 Breast Disease Center, Zhong-Da Hospital, Southeast University, Nanjing, China; 4 Department of General Surgery, Zhong-Da Hospital, Southeast University, Nanjing, China

**Keywords:** Adipocytes, PLOD2, IL-6, Leptin, Breast Cancer, Metastasis

## Abstract

**Background:**

Adipocytes make up the major component of breast tissue, accounting for 90% of stromal tissue. Thus, the crosstalk between adipocytes and breast cancer cells may play a critical role in cancer progression. Adipocyte-breast cancer interactions have been considered important for the promotion of breast cancer metastasis. However, the specific mechanisms underlying these interactions are unclear. In this study, we investigated the mechanisms of adipocyte-mediated breast cancer metastasis.

**Methods:**

Breast cancer cells were cocultured with mature adipocytes for migration and 3D matrix invasion assays. Next, lentivirus-mediated loss-of-function experiments were used to explore the function of lysyl hydroxylase (PLOD2) in breast cancer migration and adipocyte-dependent migration of breast cancer cells. The role of PLOD2 in breast cancer metastasis was further confirmed using orthotopic mammary fat pad xenografts in vivo. Clinical samples were used to confirm that PLOD2 expression is increased in tumor tissue and is associated with poor prognosis of breast cancer patients. Cells were treated with cytokines and pharmacological inhibitors in order to verify which adipokines were responsible for activation of PLOD2 expression and which signaling pathways were activated in vitro.

**Results:**

Gene expression profiling and Western blotting analyses revealed that PLOD2 was upregulated in breast cancer cells following coculture with adipocytes; this process was accompanied by enhanced breast cancer cell migration and invasion. Loss-of-function studies indicated that PLOD2 knockdown suppressed cell migration and disrupted the formation of actin stress fibers in breast cancer cells and abrogated the migration induced by following coculture with adipocytes. Moreover, experiments performed in orthotopic mammary fat pad xenografts showed that PLOD2 knockdown could reduce metastasis to the lung and liver. Further, high PLOD2 expression correlated with poor prognosis of breast cancer patients. Mechanistically, adipocyte-derived interleukin-6 (IL-6) and leptin may facilitate PLOD2 upregulation in breast cancer cells and promote breast cancer metastasis in tail vein metastasis assays. Further investigation revealed that adipocyte-derived IL-6 and leptin promoted PLOD2 expression through activation of the JAK/STAT3 and PI3K/AKT signaling pathways.

**Conclusions:**

Our study reveals that adipocyte-derived IL-6 and leptin promote PLOD2 expression by activating the JAK/STAT3 and PI3K/AKT signaling pathways, thus promoting breast cancer metastasis.

**Electronic supplementary material:**

The online version of this article (10.1186/s12964-018-0309-z) contains supplementary material, which is available to authorized users.

## Background

The tumor microenvironment plays a vital role in the initiation and progression of many cancers [1]. Adipocytes comprise approximately 90% of breast tissue. In breast cancer, the crosstalk between cancer cells and “cancer associated adipocytes” (CAAs) promotes breast cancer progression and metastasis [2, 3]. Recent research suggests that adipocytes can act as an endocrine organ, secreting several signaling molecules, such as chemokines and adipokines. Adipocyte-derived factors, such as leptin, interleukin-6 (IL-6), adiponectin, tumor necrosis factor (TNF-α), monocyte chemotactic protein-1 (MCP-1) and endotrophin (ETP), function within the tumor microenvironment to promote tumor progression [4–6]. These adipokines activate several signaling networks associated with migration, proliferation, angiogenesis, fibrosis and apoptosis, including the JAK/STAT, AKT and ERK1/2 signaling pathways, which are frequently activated in tumor tissues [7, 8].

Lysyl hydroxylases are encoded by distinct procollagen-lysine, 2-oxoglutarate 5-dioxygenase (*PLOD*) genes. These enzymes trigger the hydroxylation of collagen lysine residues prior to the formation of triple helical pro-collagen molecules [9, 10]. Over the last several years, PLOD2 deregulation has been observed in sarcoma, breast cancer, glioma, lung cancer and cervical cancer [10–14]. Additional studies have indicated that HIF-1α enhances expression of PLOD2, which in turn promotes sarcoma metastasis. Breast and lung cancers with high PLOD2 expression display enhanced rates of migration and metastasis. Knockdown of PLOD2 significantly inhibits cancer cell migration and invasion [15, 16]. Previous studies have demonstrated that the PI3K/AKT signaling pathway is involved in the regulation of PLOD2 expression [17]. In addition, it has been shown that adipocyte-derived adipokines act as profibrogenic molecules [18–20]. Additional research has reported that paracrine signals from cancer-associated fibroblasts enhance PLOD2 expression in lung cancer [13]. Therefore, these studies indicate the potential existence of an intimate crosstalk between adipokines and PLOD2.

However, little is known about how adipocyte-derived factors affect the expression of PLOD2. In this study, we investigate the function of PLOD2 in breast cancer and explore the underlying adipocyte-regulated mechanisms responsible for promoting PLOD2 expression. We used a coculture system to identify which adipokines are involved in PLOD2 expression. Through qRT-PCR analyses and RNAi knockdown experiments, we identified lysyl hydroxylase (PLOD2) as a new player in adipocyte-mediated migration. Furthermore, breast cancer cells cocultured with adipocytes or treated with either IL-6 or leptin displayed increased PLOD2 expression and activated JAK/STAT3 and AKT signaling pathways. Adipocyte-stimulated migration of breast cancer cells toward the lungs was impeded by treatment with a murine IL-6 blocking antibody or depletion of OBR. Inhibition of STAT3 and AKT activity using the pharmacological inhibitors ruxolitinib and LY294002, respectively, decreased PLOD2 expression. Taken together, these findings provide new insights into the connections between adipokines and PLOD2 in breast cancer and support a crucial role for PLOD2 in human breast cancer metastasis. Furthermore, excessive accumulation of fat may contribute to the higher expression of PLOD2 and correlation with breast cancer patient poor prognosis.

## Methods

### Cell culture

The MDA-MB-231 and MDA-MB-468 cell lines were obtained from American Type Culture Collection (Manassas, VA, USA) and cultured in Dulbecco’s Modified Eagle Medium (DMEM, 12100046, Thermo Fisher Scientific, Waltham, MA, USA). The culture medium contained 10% fetal bovine serum (FBS), penicillin (50 U/mL), and streptomycin (50 U/mL). MDA-MB-231 and MDA-MB-468 were used within 6 months of passaging after purchase. 3 T3-L1 preadipocyte cells, SK-BR-3 human breast cancer cells and 293 T embryonic kidney cells were purchased from The Shanghai Institute of Life Science, Chinese Academy of Science. 3 T3-L1 cells were maintained in Dulbecco’s Modified Eagle Medium (DMEM) containing 10% fetal calf serum (FCS), penicillin (50 U/mL), and streptomycin (50 U/mL). SK-BR-3 and 293 T cells were cultured in DMEM/F12 with 10% FBS, penicillin (50 U/mL) and streptomycin (50 U/mL). All cells were maintained in a humidified 5% CO2 atmosphere at 37 °C.

The 3 T3-L1 preadipocyte cell line was differentiated as previously described [21]. At 90% confluency, 3 T3-L1 cells were detached with trypsin-EDTA (SunShine Biotechnology) and seeded in 6-well plates (WHB). After reaching confluency, differentiation was induced by incubating cells in DMEM containing 10% FBS, 0.5 mM IBMX (Sigma), 10 μg/mL insulin (Sigma) and 1 μM dexamethasone (Sigma) for a total of 6 days, with a media change after 3 days. Then, the medium was replaced with an adipocyte-maintaining medium comprised of DMEM enriched with 10% FBS and 10 μg/mL insulin for a total of 6 days, with a media change after 3 days. Oil Red O staining was performed as described previously [21]. Human preadipocyte cells were purchased from ScienCell Research Laboratory and were cultured and differentiated identically to the 3 T3-L1 preadipocytes.

### Coculture and migration assays

After 12 days of adipocyte induction, the cells were cocultured with MDA-MB-231 and MDA-MB-468 human breast cancer cells using a Transwell culture system (0.4 μm pore size, Corning). 3.0 × 10^5^ MDA-MB-231 and MDA-MB-468 cells were seeded in the top chamber. Breast cancer cells were cocultured with adipocytes for 3 h, 6 h, 12 h, 24 h, 48 h and 72 h. MDA-MB-231 and MDA-MB-468 cells were cultured alone as controls and were evaluated at the same time points. To evaluate the effects of adipocyte coculture on breast cancer tumor cell migration, after 3 days of coculture with MDA-MB-231 and MDA-MB-468 cells, tumor cells were used to conduct migration assays using 24-well Boyden chambers containing inserts (8 μm, pores, BD Biosciences, USA). 5 × 10^4^ cells were cultured in the upper wells of Transwell chambers and allowed to migrate toward 10% FBS in the bottom wells. Similar migration assays were conducted with monocultured tumor cells. After 12 h, migrated cells were stained with Diff-Stain Set (Jiancheng), photographed, and counted using ImageJ software.

### Spheroid invasion assays in Matrigel matrix

The hanging-drop method has been previously used to generate spheroids [22, 23]. MDA-MB-231 and MDA-MB-468 breast cancer cells were detached following 3 days of either monoculture or coculture with adipocytes. Drops of 1000 tumor cells each were formed using 20 μL medium supplemented with methylcellulose (20%). Cells were incubated as droplets (25 μL) for 48 h to ensure multicellular aggregation. For the spheroid invasion assays, hybrid aggregates were implanted into Matrigel and cultured for 7 days, after which time tumor cell invasion was apparent.

### Colony formation assays

Six-well plates were seeded at 1000 cells/well and then cells were cultured in complete medium for almost 20 days. Cells were fixed with 4% formaldehyde and stained with 0.5% crystal violet. Colonies were counted using Image-Pro Plus 6.0.

### Recombinant human IL-6 and leptin proteins

MDA-MB-231 and MDA-MB-468 cells were treated with human recombinant IL-6 protein (5 ng/mL, R&D Systems) and human recombinant leptin protein (50 ng/mL, GeneScript).

### Immunofluorescence and immunohistochemistry

Tumor cells were seeded on coverslips and either monocultured or cocultured for 3 days with adipocytes or treated with IL-6 and leptin. Cells were fixed in 4% paraformaldehyde for 30 min at room temperature and then permeabilized with 0.3% Triton X-100 in 3% BSA for 15 min at room temperature [24]. Then, 3% bovine serum albumin in PBS was used to block cells for 1 h. Next, cells were incubated with a primary antibody against type I collagen or P-STAT3 overnight at 4 °C. Cells were incubated with the appropriate secondary antibody and Hoechst stain for 1 h at room temperature, with extensive washing between every step. Images were captured using a confocal microscope.

Paraffin sections were deparaffinized according to a standard protocol. Antigen retrieval was performed in citrate buffer for 2 min at 100 °C. Sections were blocked with 5% BSA and then incubated with a PLOD2 antibody overnight at 4 °C. Sections were then incubated with a biotin-labeled rabbit anti-goat antibody for 30 min at room temperature. Sections were visualized with DAB and counterstained with hematoxylin.

### RNA extraction and quantitative real time PCR

Total cellular RNA was isolated with TRIzol® Reagent (Vazyme) and reverse transcribed with the RevertAid™ First Strand cDNA Synthesis Kit (Takara). qRT-PCR assays were performed to analyze relative mRNA levels using a SYBR Green-based system (Applied Biosystems). The amount of mRNA for each gene was normalized to the internal control (18S or GAPDH). The sequences of the primers used in this study are provided in Additional file 1: Table S1.

### Dot hybridization for the detection of IL-6 and leptin secretion by adipocytes following coculture with breast cancer cells

Conditioned medium was collected from 3 T3-L1 cells, adipocytes and adipocytes cocultured with breast cancer cells. 20 μL of conditioned medium was added to a PVDF membrane containing 1 μg of protein. The membrane was allowed to dry and was then blocked with 5% bovine serum albumin for 1 h at room temperature. Next, the membrane was incubated with a primary antibody against IL-6 or leptin overnight at 4 °C. Then, the membrane was incubated with the appropriate secondary antibody for 1 h at room temperature, with extensive washing between every step. Membranes were analyzed using an Enhanced Chemiluminescence (ECL) detection system.

### Western blotting

Cells were lysed in cell lysis buffer to extract total proteins. Proteins were then separated by SDS-PAGE and transferred to a PVDF membrane. Membranes were analyzed using an Enhanced Chemiluminescence (ECL) detection system. The antibodies used in this study are presented in Additional file 1: Table S2.

### Transfection and generation of stable cells

shRNAs targeting human PLOD2 (SHC [V2LHS_131378], SHD [V3LHS_306074]) as well as a scrambled shRNA were received from the shRNA and ORFeome Core at the MD Anderson Cancer Center. Lentiviruses were generated with either shRNA or cDNA that had been packaged in 293 T cells using pMD2.G and psPAX2 plasmids. 48 h later, conditioned medium was collected from 293 T cells and used to infect target cells. Stable cell lines were generated by selection with puromycin (10 μg/mL).

### Transient transfection with siRNAs

Small-interfering RNAs (siRNAs) against OBR were synthesized by GenePharma (Shanghai, China). The sequences of the OBR and negative control siRNAs are presented in Additional file 1: Table S3. Prior to transfection, MDA-MB-231 and MDA-MB-468 breast cancer cells were plated in 6-well plates at 50% confluency. OBR and negative control siRNAs were transfected into cells using Lipofectamine 2000 Reagent (Life Technologies, USA) according to the manufacturer’s protocol. Cells were collected after 48–72 h for qRT-PCR, Western blotting or coculture experiments.

### Orthotopic xenograft tumor model

Five to six week old female NOD SCID mice (MARC, Nanjing University, China) were randomly divided into groups (six mice per group). All animal procedures were approved by the Experimental Animal Care Commission of China Pharmaceutical University. Mice were anesthetized by isoflurane inhalation, then MDA-MB-231-SCR and MDA-MB-231-SHC human breast cancer cells (2 × 10^6^ cells per mouse) were orthotopically injected into the inguinal mammary fat pad. Each injection contained 50 μL cell suspension, including 25 μL Matrigel (Corning). Tumor volume was measured using calipers and calculated as V = (L × W^2^)/2. After 9 weeks, tumor metastasis was assessed by bioluminescent imaging on the Xenogen In Vivo Imaging System (IVIS, Caliper Life Science, Hopkinton, MA). Mice were then sacrificed and lungs and livers were formalin-fixed and paraffin-embedded for hematoxylin and eosin staining. Liver metastases were quantified in five random low power fields per group and are presented as the mean ± s.d.

### Tail vein metastasis assays

MDA-MB-231 cells were monocultured, cocultured with adipocytes, cocultured with adipocytes in the presence of a murine IL-6 blocking antibody, or cocultured with adipocytes following OBR depletion. After 3 days, these cells were harvested, centrifuged, and gently resuspended in Cell Tracker™ blue (CMTPX) (Thermo Fisher Scientific, C2925). This reagent was prewarmed (at room temperature) and diluted to a final concentration of 10 μM according to the manufacturer’s instructions. The cells were then incubated at 37 °C for 30 min, centrifuged again and resuspended in serum free media to a concentration of 2.5 × 10^5^ cells per 100 μL. A total of 5 × 10^5^ cells per 200 μL were injected into the tail vein of each NOD SCID mouse. After 2 weeks, mice were sacrificed and lungs were isolated and sectioned for histological studies by fluorescence microscopy. The rest of the lungs were formalin-fixed and paraffin-embedded for hematoxylin and eosin staining. Lungs from different treatment groups were photographed under a microscope to compare the distribution (and, thus, metastatic capacity) of the cells in the lungs.

### Database

Correlations between relapse-free survival (RFS) of breast cancer patients and PLOD2 expression were analyzed using the Kaplan-Meier plotter (http://kmplot.com/analysis/) [25]. The expression level of PLOD2 in triple negative breast cancer (TNBC) and non-triple negative breast cancer (non-TNBC) were analyzed using The Cancer Genome Atlas (TCGA) database.

### Tumor tissue microarray

Human breast cancer specimens were obtained from the Shanghai Outdo Biotech Co., Ltd., China. 150 breast cancer patients (ages 31–82) were selected for this experiment. PLOD2, GP130 and OBR expression were detected by immunochemistry.

### Statistical analysis

Mouse body weights and tumor volumes were analyzed by two-way ANOVA. KM curves were plotted and log-rank tests were used to determine statistical significance between the survival rates of patients with high and low PLOD2 expression. The remaining results are presented as the means ± SD from triplicate experiments. Student’s t-test (two-tailed, unpaired) or one-way ANOVA (three-tailed or more, unpaired) was used to compare control and treatment groups. ^*^represents statistical significance with *P* < 0.05, ^**^represents statistical significance with *P* < 0.01.

## Results

### PLOD2 is upregulated during adipocyte-stimulated migration and invasion

**Fig. 1:**
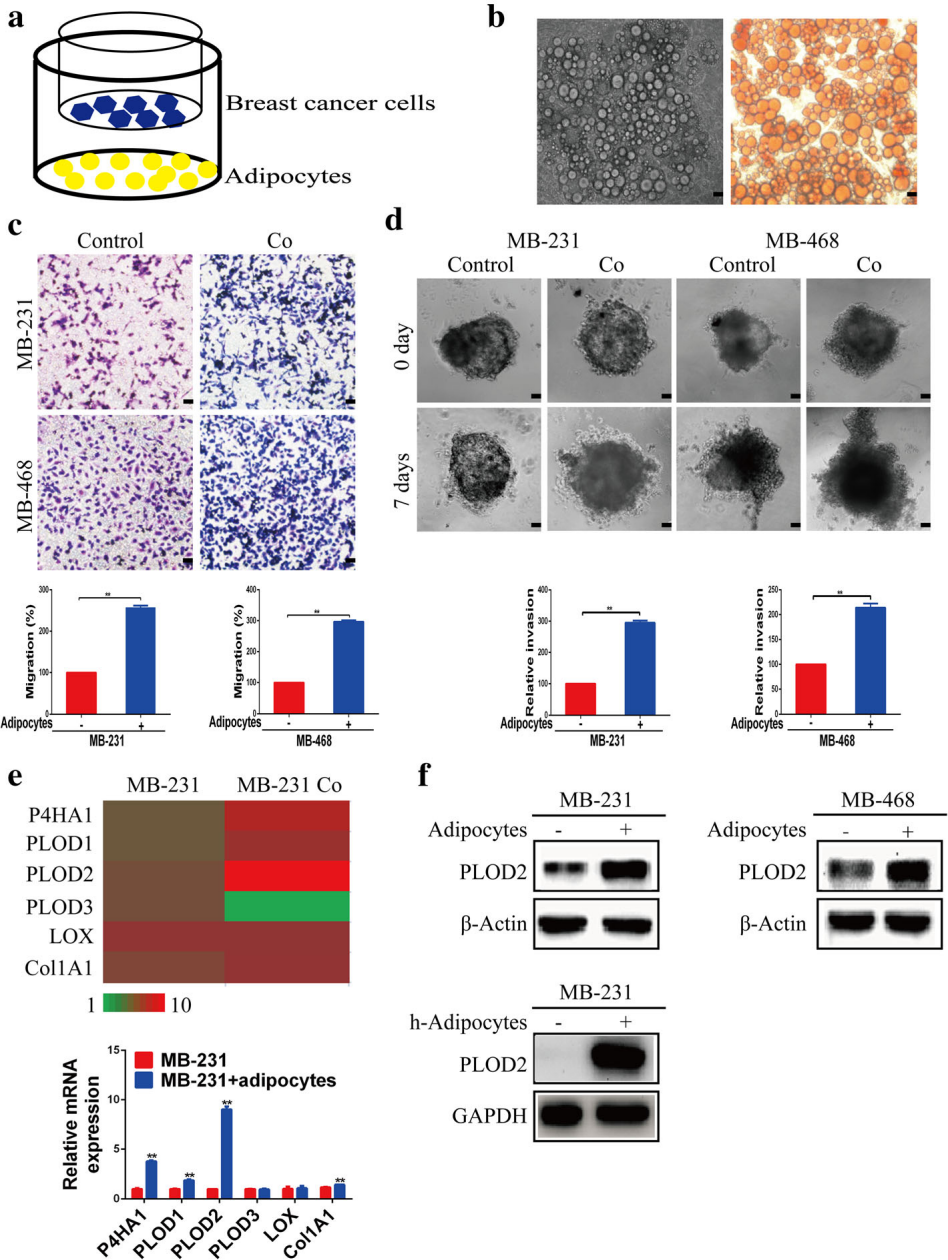
PLOD2 is upregulated during adipocyte-driven migration and invasion in breast cancer cells. **a** Schematic diagram of the coculture system. Adipocytes were seeded in the bottom layer and breast cancer cells were seeded in the upper layer of the Transwell culture system. Cells were then cocultured for 3 days. **b** Mature adipocytes were stained with Oil Red O to confirm differentiation efficiency. **c**-**d** MDA-MB-231 (MB-231) or MDA-MB-468 (MB-468) cells were cocultured with adipocytes (Co) or monocultured (Control) for 3 days. Then, cancer cells were used for migration (**c**) and 3D matrix invasion assays (**d**). Error bars represent means ± SD. ^**^P < 0.01. Scale bars, 100 μm. **e** Relative mRNA expression levels of P4HA1, PLOD1, PLOD2, PLOD3, LOX and Col1A1 in MDA-MB-231 breast cancer cells cocultured with adipocytes or monocultured for 3 days. Error bars represent means ± SD. ^**^P < 0.01. **f** MDA-MB-231 (MB-231), MDA-MB-468 (MB-468) breast cancer cells were cocultured with adipocytes or monocultured. After 3 days, cells were harvested and PLOD2 protein expression was detected by Western blotting. MDA-MB-231 (MB-231) cancer cells were cocultured with human adipocytes. After 3 days, cells were harvested and PLOD2 protein expression was detected by Western blotting

We first established an adipocyte-breast cancer cell coculture system, shown in Fig. 1a, where adipocytes were seeded in the bottom layer and breast cancer cells were seeded in the upper layer of a Transwell culture system. This allows for crosstalk between the cells through diffusible factors. The murine 3 T3-L1 preadipocytes were differentiated as previously described [21]. As shown in Fig. 1b, adipocyte maturation was confirmed by Oil Red O staining. Following 3 days of coculture, MDA-MB-231 and MDA-MB-468 cells were trypsinized and used in migration assays. Our results showed that migration was increased in cells cocultured for 3 days with adipocytes, compared to tumor cells grown alone (Fig. 1c). Next, we explored breast cancer cell invasion using a spheroid invasion model in a Matrigel matrix. As shown in Fig. 1d, MDA-MB-231 and MDA-MB-468 breast cancer cells cocultured with adipocytes exhibited increased invasion compared with spheroids generated from monocultured cancer cells. Taken together, these results suggest that adipocytes promote breast cancer migration and invasion.

Next, we explored how adipocyte promote breast cancer cell migration and invasion. Fischbach and colleagues have revealed that obesity-induced interstitial fibrosis promotes breast tumorigenesis [26]. Previous studies have revealed that adipokines are associated with inflammation and fibrosis [27]. For example, leptin can induce liver fibrosis through the activation of hepatic stellate cells (HSCs) [28]. Children with cystic fibrosis display high serum levels of adipokines, suggesting that adipokines may be involved in fibrotic disease [29]. Over-deposition of collagen is the main cause of fibrosis, and aberrant PLOD2 expression contributes to the progression of collagen-related diseases such as fibrosis and cancer [30]. Therefore, we speculated that a link might exist between adipokines and PLOD2. We first compared collagen distribution patterns in breast cancer cells that were either monocultured or cocultured with adipocytes. The results showed that collagen deposition was significantly increased upon coculture with adipocytes (Additional file 2: Figure S1a). qRT-PCR analysis demonstrated that expression of collagen biogenesis-associated genes (P4HA1, PLOD1, PLOD2) were elevated after coculture with adipocytes. Of these genes, PLOD2 was significantly increased, as shown in Fig. 1e. PLOD2 protein expression was also increased in MDA-MB-231 and MDA-MB-468 breast cancer cells cocultured with adipocytes (Fig. 1f). Similar results were obtained by coculturing MDA-MB-231 and SK-BR-3 breast cancer cells with or without human mammary adipocytes (Fig. 1f, Additional file 2: Figure S1b). Taken together, these results suggest that PLOD2 upregulation may stimulate the enhanced migration of breast cancer cells following coculture with adipocytes.

### PLOD2 knockdown attenuates breast cancer migration and metastasis in vitro and in vivo

**Fig. 2:**
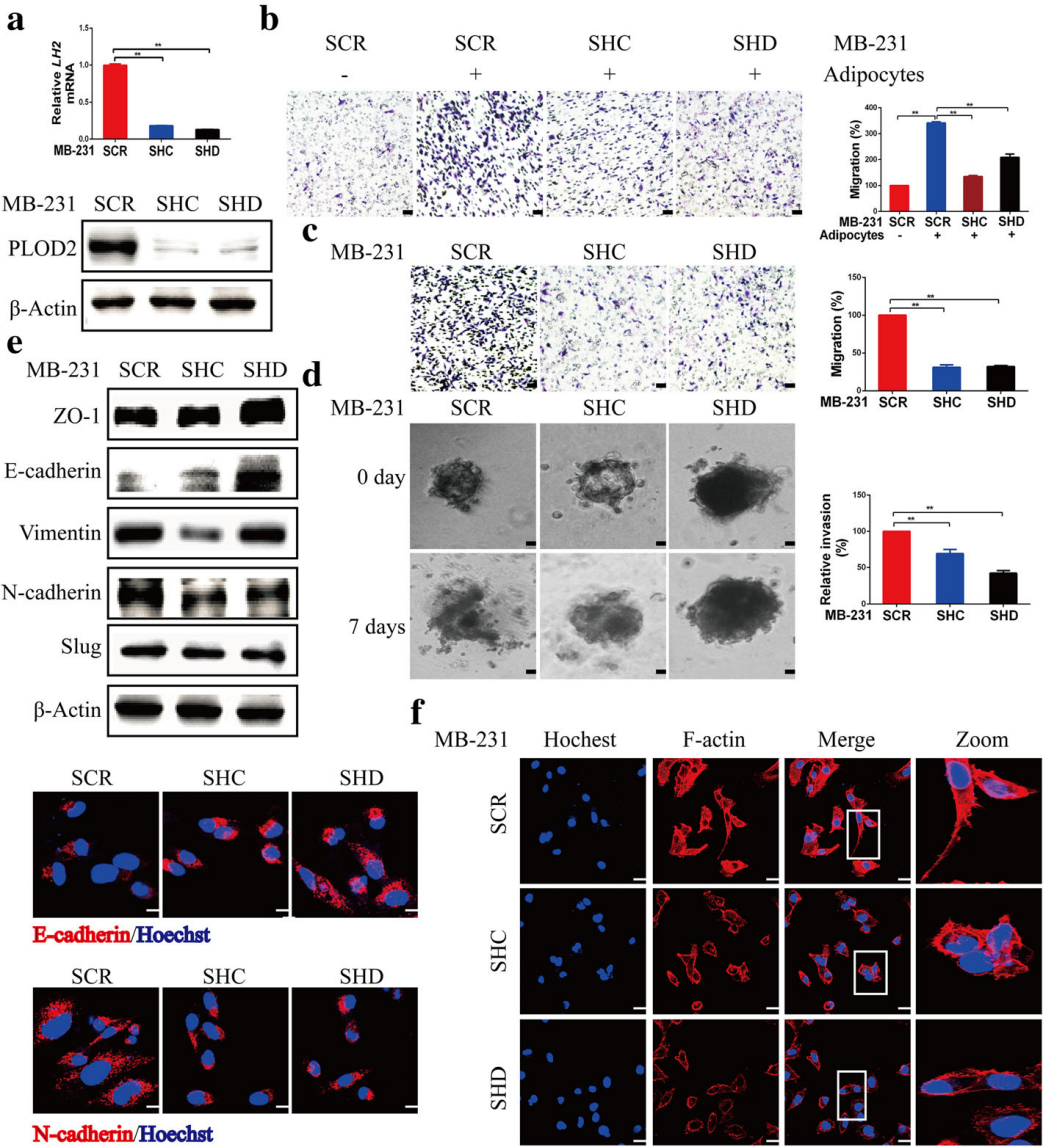
PLOD2 knockdown attenuates breast cancer cell migration in vitro. **a** PLOD2 was knocked down using two independent short hairpin RNAs (SHC and SHD) in MDA-MB-231 (MB-231) cells. qRT-PCR and Western blotting were used to detect PLOD2 expression in scramble (SCR) and PLOD2-knockdown cells (SHC, SHD). Error bars represent means ± SD. ^**^P < 0.01. **b** Scramble (SCR) and PLOD2-knockdown cells (SHC, SHD) were cocultured in the presence or absence of adipocytes. After 3 days, cells were used for migration assays. Error bars represent means ± SD. ^**^P < 0.01. Scale bars, 100 μm. **c**-**d** Scramble (SCR) and PLOD2-knockdown cells (SHC, SHD) were evaluated by migration (**c**) and 3D matrix invasion assays (**d**). Error bars represent means ± SD. ^**^P < 0.01. Scale bars, 100 μm. **e** Expression levels of E-cadherin, N-cadherin, Vimentin, ZO-1 and Slug were detected by Western blotting in scramble (SCR) and PLOD2-knockdown cells (SHC, SHD). Immunofluorescence staining for E-cadherin (red) and N-cadherin (red) in scramble (SCR) and PLOD2-knockdown cells (SHC, SHD). Nuclei were stained with Hoechst (blue). Scale bars, 20 μm. **f** Immunofluorescence staining for F-actin (red) in scramble (SCR) and PLOD2-knockdown cells (SHC, SHD). Nuclei were stained with Hoechst (blue). Scale bars, 20 μm

To investigate the role of PLOD2 during adipocyte-dependent migration and metastasis of breast cancer cells in vitro, we examined the effects of PLOD2 knockdown in MDA-MB-231 cells on migration, invasion, EMT and actin stress fiber formation. Knockdown of PLOD2 was achieved using lentiviral shRNAs (PLOD2-SHC and SHD) and was confirmed by qRT-PCR and Western blotting (Fig. 2a). PLOD2 depletion significantly inhibited the migration of MDA-MB-231 tumor cells cocultured with adipocytes (Fig. 2b). Moreover, PLOD2 depletion significantly reduced MDA-MB-231 cell migration and 3D Matrigel matrix invasion in the absence of adipocytes (Fig. 2c, d). Notably, knockdown of PLOD2 had no detectable effect on MDA-MB-231 cell proliferation in colony formation assays (Additional file 3: Figure S2a). We further examined the expression levels of some EMT-associated regulators in MDA-MB-231 cells by Western blotting and immunofluorescence. Depletion of PLOD2 in MDA-MB-231 cells either abrogated or increased the expression of N-cadherin, vimentin, slug, E-cadherin and zona occludens (ZO-1) (Fig. 2e). Furthermore, immunofluorescence experiment confirmed that shRNA-mediated PLOD2 knockdown in MDA-MB-231 cells increased the expression of E-cadherin and decreased the expression of N-cadherin (Fig. 2e). The filamentous actin (F-actin) cytoskeleton is vital for cell migration. Therefore, we explored whether PLOD2 knockdown altered the arrangement of cellular F-actin. We found that knockdown of PLOD2 in MDA-MB-231 cells resulted in disruption and loss of actin stress fibers (Fig. 2f). These results strongly suggest that PLOD2 is involved in breast cancer cell metastasis.

**Fig. 3:**
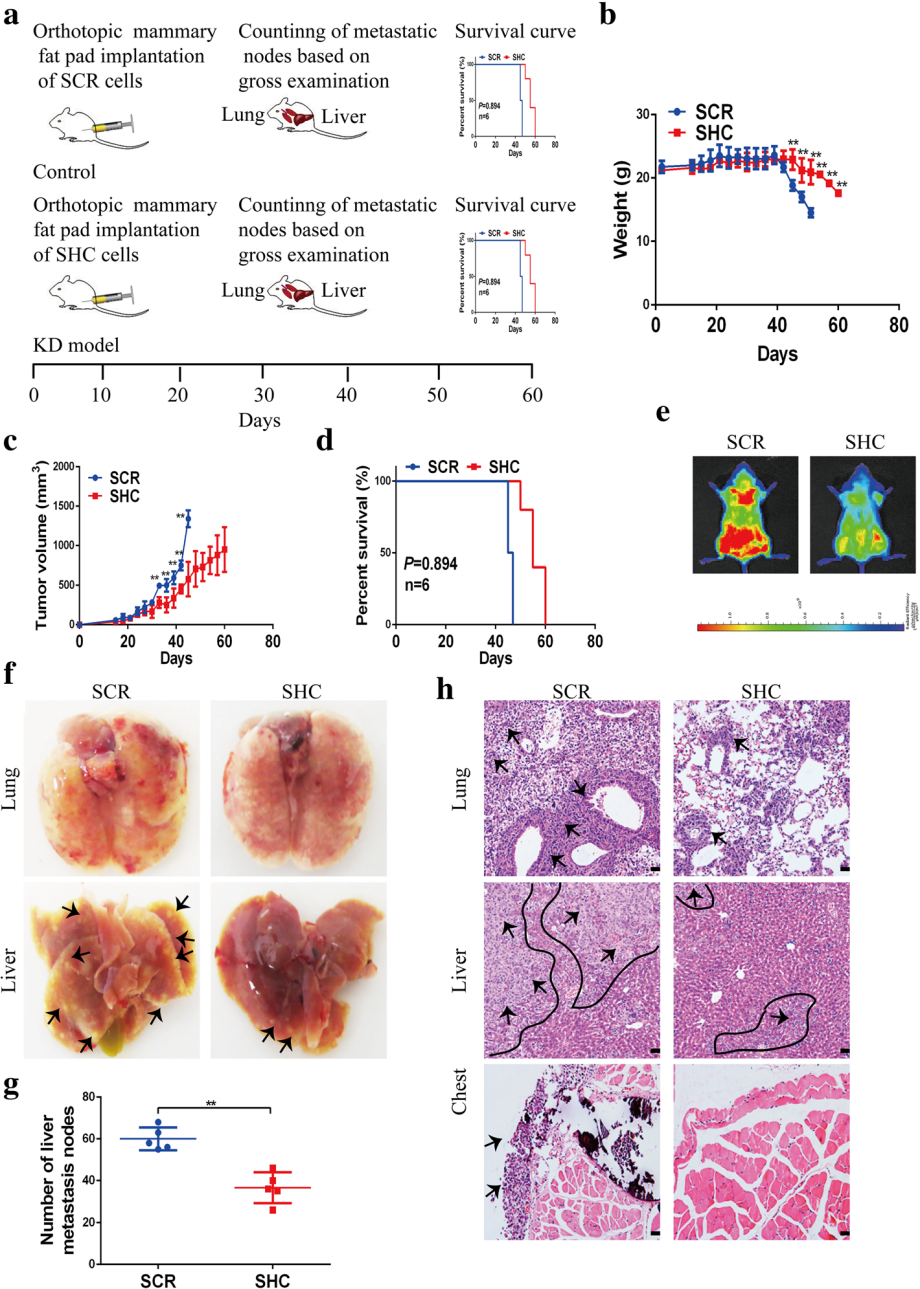
Knockdown of PLOD2 suppresses metastasis in vivo. **a** Schematic diagram of orthotopic mammary fat pad injections of scramble (SCR) or PLOD2-knockdown breast cancer cells (SHC). **b** Knockdown of PLOD2 delayed weight loss and improved quality of life in orthotopically injected mice. Error bars represent means ± SD. (*n* = 6/group) ^**^P < 0.01. **c** Graph of primary tumor volumes. PLOD2-deficient MDA-MB-231 tumors were smaller than SCR MDA-MB-231 tumors. Error bars represent means ± SD. (*n* = 6/group) ^**^P < 0.01. d Kaplan-Meier survival curves for orthotopically injected mice. **e** In vivo metastasis assays with SCR or PLOD2-knockdown MDA-MB-231 cells. Metastatic burden was detected by bioluminescent imaging (BLI) 56 days after injection. The color scale depicts the photon flux (photos per second) emitted from the metastatic cells. **f**-**g** Knockdown of PLOD2 decreased the number of metastatic tumor foci in the lung and liver. Mice were euthanized 56 days after injection and lungs and livers were excised (**f**). Metastatic nodes in the liver were counted based on gross examination (**g**). Error bars represent means ± SD. (*n* = 6/group) ^**^P < 0.01. **h** The metastatic nodes in the lungs, liver and chest were further detected by hematoxylin and eosin (H&E) staining. Arrows indicate metastatic colonization. Scale bars, 100 μm

Based on these findings, we next wanted to determine whether PLOD2 deficiency regulates tumor cell metastatic capacity in vivo. To address this question, we orthotopically injected breast cancer cells into the mammary fat pads of NOD SCID mice. The experimental procedure for these in vivo studies is shown in Fig. 3a. PLOD2-deficient MDA-MB-231 tumors were smaller and metastasized less frequently to the lung and liver (Fig. 3c, f, g, h). Moreover, bioluminescent imaging (BLI) of orthotopically injected mice confirmed that PLOD2 knockdown significantly reduced the metastatic burden in the lung and liver (Fig. 3e). Further, PLOD2 knockdown correlated with decreased tumor weight (Additional file 3: Figure S2b). Additionally, compared with the control group, metastatic nodes in the chest wall were markedly decreased in mice bearing PLOD2-silenced tumors (Fig. 3h). Moreover, knockdown of PLOD2 improved the quality of life and prolonged survival of injected mice compared with mice implanted with MDA-MB-231 tumors transfected with the scrambled shRNA (Fig. 3b, d). Immunohistochemical staining for PLOD2 confirmed that knockdown of PLOD2 decreased tumor PLOD2 protein expression (Additional file 3: Figure S2c). Moreover, immunohistochemical staining for PLOD2 in normal tissues and metastatic nodules indicated that PLOD2 was positively expressed in normal lung and liver tissues and metastatic nodules (Additional file 3: Figure S2d), while there were no difference between normal tissues and metastatic nodules. Therefore, we speculated that maybe the high expression of PLOD2 in lung and liver normal tissues induced the formation of pre-metastatic lesions, which was conductive to the metastasis of breast cancer. Taken together, these results suggest that PLOD2 promotes breast cancer metastasis in vivo.

### Adipocyte-derived IL-6 and leptin regulate PLOD2 expression

**Fig. 4:**
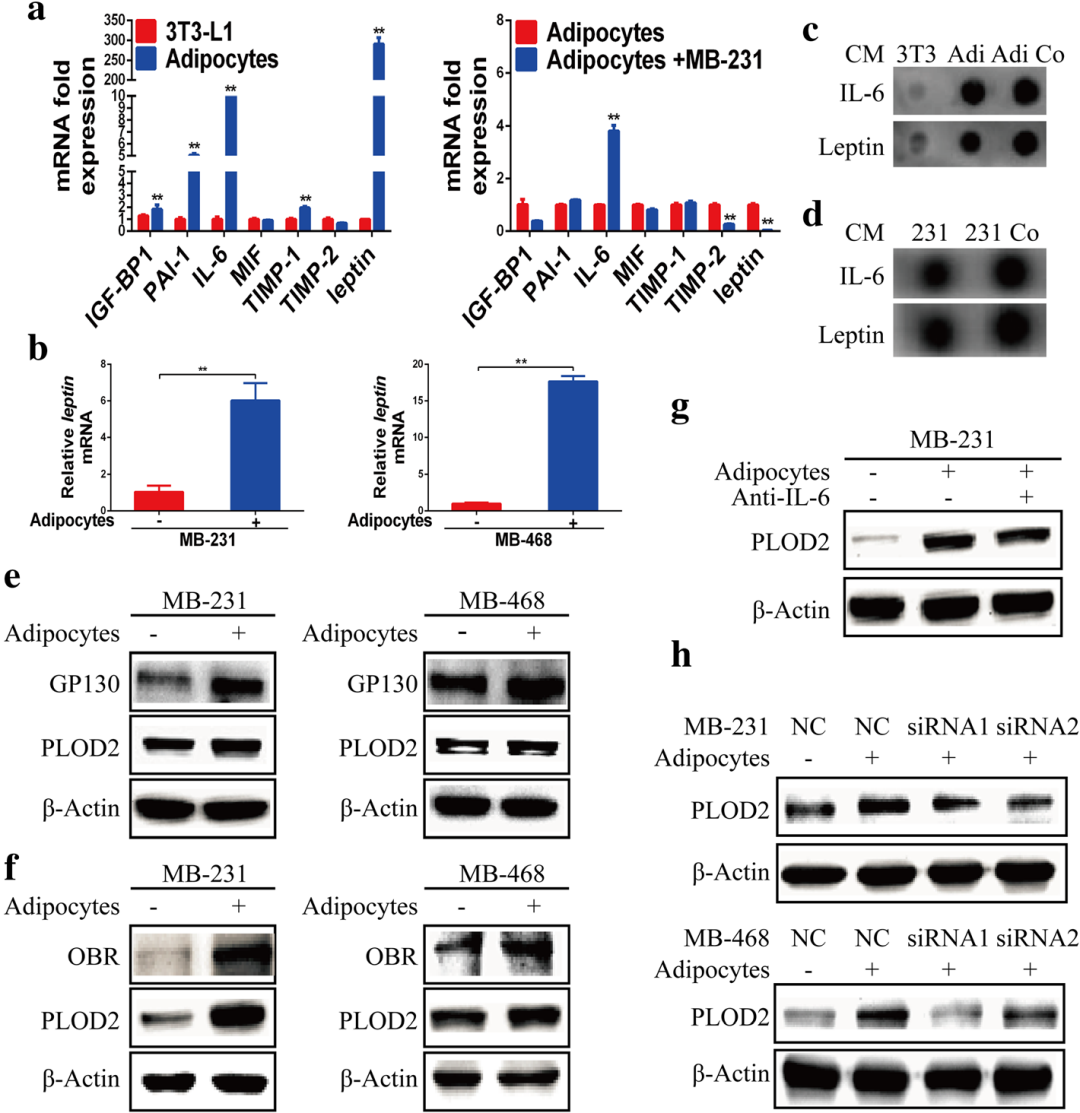
Adipocyte-derived IL-6 and leptin regulate PLOD2 expression. **a** qRT-PCR analysis of the relative expression levels of IGF-BP1, PAI-1, IL-6, MIF, TIMP-1, TIMP-2 and Leptin in 3 T3-L1 preadipocytes, adipocytes and adipocytes cocultured with MDA-MB-231 (MB-231) breast cancer cells. Error bars represent means ± SD. ^**^P < 0.01. **b** qRT-PCR analysis of relative leptin expression levels in MDA-MB-231 (MB-231) and MDA-MB-468 (MB-468) cells that were either monocultured or cocultured with adipocytes. **c** Dot hybridization analysis of IL-6 and leptin secretion in 3 T3-L1 preadipocytes, adipocytes and adipocytes cocultured with MDA-MB-231 (MB-231) breast cancer cells. **d** Dot hybridization analysis of IL-6 and leptin secretion in MDA-MB-231 (MB-231) cells and MDA-MB-468 (MB-468) cells cocultured with adipocytes. **e** MDA-MB-231 (MB-231) and MDA-MB-468 (MB-468) breast cancer cells were monocultured or cocultured with adipocytes. After 3 days, tumor cells were collected and GP130 and PLOD2 protein expression was examined by Western blotting. **f** MDA-MB-231 (MB-231) and MDA-MB-468 (MB-468) breast cancer cells were monocultured or cocultured with adipocytes. After 3 days, tumor cells were collected and OBR and PLOD2 protein expression was examined by Western blotting. **g** MDA-MB-231 (MB-231) breast cancer cells were monocultured or cocultured with adipocytes. A blocking antibody directed against IL-6 was added to the culture medium of experimental cells. After 3 days, tumor cells were harvested to detect PLOD2 protein expression. **h** PLOD2 protein expression was analyzed by Western blot in MDA-MB-231 (MB-231) and MDA-MB-468 (MB-468) breast cancer cells transiently transfected with either vector or one of two siRNAs against the leptin receptor (OBR) and then either monocultured or cocultured with adipocytes

Based on previous findings, we speculated that the adipocyte-regulated promotion of PLOD2 expression is most likely mediated by soluble factors. To address this question, we used qRT-PCR to compare the expression of several candidate adipokines in 3 T3-L1 preadipocytes, adipocytes and adipocytes cocultured with breast cancer cells. As shown in Fig. 4a, the mRNA expression levels of leptin, IL-6, plasminogen activator inhibitor 1 (PAI-1), insulin like growth factor 1 (IGF-1), and tissue inhibitor of metalloproteinase 1 (TIMP1) were increased in adipocytes. Specifically, adipocytes expressed much higher levels of leptin and IL-6 compared with other adipokines. Expression of IL-6 increased while leptin expression decreased when adipocytes were cocultured with MDA-MB-231 breast cancer cells (Fig. 4a). However, IL-6 expression was not increased when adipocytes were cocultured with MDA-MB-468 cells (Additional file 4: Figure S3a). In cocultured adipocytes, leptin mRNA expression was significantly decreased, while in cocultured breast cancer cells, leptin mRNA expression was significantly increased (Fig. 4b). Therefore, we speculated that coculture activates leptin expression in MDA-MB-231 breast cancer cells, forming a feedback loop. Based on our qRT-PCR results, we next detected levels of secreted IL-6 and leptin in the medium of 3 T3-L1 preadipocytes, adipocytes and adipocytes cocultured with breast cancer cells. The secretion of IL-6 and leptin was increased in the supernatant of adipocytes cocultured with MDA-MB-231 and MDA-MB-468 breast cancer cells compared with 3 T3-L1 preadipocytes and adipocytes (Fig. 4c, Additional file 4: Figure S3b). Moreover, the secretion of IL-6 and leptin was significantly higher in the supernatant of MDA-MB-231 and MDA-MB-468 breast cancer cells after adipocyte coculture as compared with MDA-MB-231 and MDA-MB-468 cells grown alone (Additional file 4: S3c Fig. 4d). Collectively, our results clearly show that adipocyte coculture stimulates overexpression of adipokines, such as IL-6 and leptin.

To further verify that adipokines contribute to adipocyte-mediated effects, we investigated the expression of PLOD2 in breast cancer cells cultured in conditioned medium (CM) obtained from preadipocytes, adipocytes, or adipocytes previously grown in the presence of cancer cells. Strikingly, PLOD2 expression increased in MDA-MB-231 and MDA-MB-468 cells following culturing of these cells in CM obtained from adipocytes and CM obtained from adipocytes previously grown in the presence of cancer cells. Meanwhile, CM from preadipocytes had little effect on the expression of PLOD2 (Additional file 4: Figure S3d). Altogether, these results confirm that adipocyte-derived soluble factors play a key role in adipocyte-induced PLOD2 expression. Further, it is likely that IL-6 and leptin are essential for the observed effects of conditioned medium on PLOD2 expression.

### IL-6/GP130 and leptin/OBR mediate adipocyte microenvironment-induced activation of PLOD2 and migration in breast cancer cells

Thus far, we have demonstrated that IL-6 and leptin are increased in adipocytes and/or adipocytes cocultured with breast cancer cells. We next explored whether the expression of IL-6 and leptin receptors, which might mediate PLOD2 expression, was increased in MDA-MB-231 and MDA-MB-468 breast cancer cells after coculture with adipocytes. GP130, a subunit of the IL-6 receptor, plays a critical role in mediating IL-6 signaling [31, 32]. Here, we demonstrated that protein levels of GP130 and PLOD2 were significantly increased in MDA-MB-231 and MDA-MB-468 breast cancer cells cocultured with adipocytes (Fig. 4e). Strikingly, PLOD2 expression decreased when MDA-MB-231 cells and SK-BR-3 cells were cocultured with adipocytes in the presence of a murine IL-6 blocking antibody (Fig. 4g, Additional file 4: Figure S3e). It is known that leptin binds to its receptor (OBR/LEPR) on the cell surface, and can thus activate a number of complex signaling cascades [33]. Thus, we next examined OBR protein levels in breast cancer cells cocultured with adipocyte. Interestingly, adipocyte coculture significantly increased the protein expression levels of both OBR and PLOD2 (Fig. 4f). Inversely, OBR depletion abolished the adipocyte-induced upregulation of PLOD2 (Fig. 4h, Additional file 4: Figure S3f). Taken together, these results indicate that adipocyte-derived IL-6 and leptin promote PLOD2 expression through activation of their respective receptors.

**Fig. 5:**
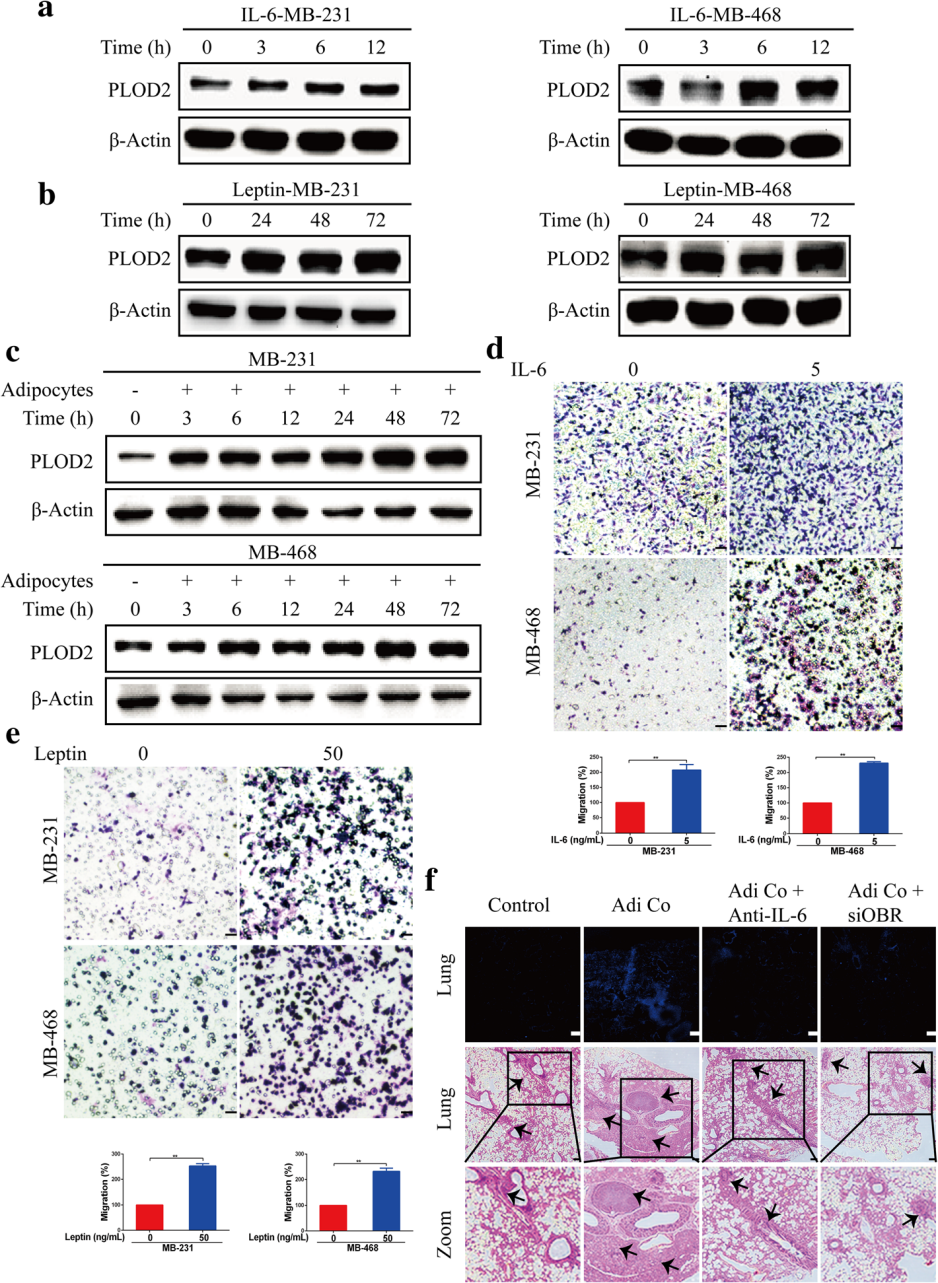
IL-6/GP130 and leptin/OBR mediate adipocyte microenvironment-induced activation of PLOD2 and migration in breast cancer. **a** PLOD2 protein expression in MDA-MB-231 (MB-231) and MDA-MB-468 (MB-468) cells treated with the indicated IL-6 concentration for 0, 3, 6, or 12 h. **b** PLOD2 protein expression in MDA-MB-231 (MB-231) and MDA-MB-468 (MB-468) cells treated with the indicated concentration of leptin for 0, 24, 48, or 72 h. **c** MDA-MB-231 (MB-231) and MDA-MB-468 (MB-468) cells were monocultured or cocultured with adipocytes for a variety of times (0 to 72 h). Tumor cells were harvested to detect PLOD2 protein expression. **d**-**e** MDA-MB-231 (MB-231) and MDA-MB-468 (MB-468) cells were treated with the indicated concentration of IL-6 (**d**) or leptin (**e**) for 12 or 72 h and then harvested to conduct migration assays. Error bars represent means ± SD. Scale bars, 100 μm. **f** MDA-MB-231 cells were monocultured, cocultured with adipocytes, cocultured with adipocytes in the presence of a murine IL-6 blocking antibody, or cocultured with adipocytes following OBR depletion. After 3 days, these cells were harvested and centrifuged, and then the cells were gently resuspended in Cell Tracker™ blue (CMTPX). A total of 5 × 10^5^ cells per 200 μL were then injected into the tail vein of each NOD SCID mouse. After 2 weeks, mice were sacrificed and lungs were isolated and sectioned for histological studies by fluorescence microscopy. Scale bars, 100 μm. Hematoxylin and eosin (H&E) staining of transverse sections of lungs from mice injected with MDA-MB-231 cells monocultured, cocultured with adipocytes, cocultured with adipocytes in the presence of a murine IL-6 blocking antibody, or cocultured with adipocytes following OBR depletion. Scale bars, 200 μm. Invasive lesions are indicated with arrows

To further confirm whether IL-6 and leptin could induce PLOD2 expression, we treated MDA-MB-231 and MDA-MB-468 breast cancer cells with recombinant human IL-6 protein. We observed that treatment with recombinant IL-6 enhanced PLOD2 expression in a time-dependent manner (Fig. 5a). Interestingly, a 3 day exposure to IL-6 had no effect on the expression of PLOD2 in MDA-MB-231 and MDA-MB-468 breast cancer cells. (Additional file 4: Figure S3 g). We therefore speculated that IL-6 promotes PLOD2 expression within a short time period. We next examined the expression of PLOD2 following treatment of MDA-MB-231 and MDA-MB-468 breast cancer cells with leptin. We observed that treatment with recombinant human leptin resulted in a significant increase in PLOD2 expression in breast cancer cells in a time-dependent manner (Fig. 5b). However, leptin had no effect on PLOD2 expression between 3 h and 24 h of exposure (Additional file 4: Figure S3 h). Therefore, leptin enhances PLOD2 expression via long-term stimulation. Combining this observation with our results showing that IL-6 enhances PLOD2 expression in the short term, we hypothesized that adipocyte coculture would increase PLOD2 expression in the short term and maintain this expression in the long term in breast cancer cells. Thus, we next examined PLOD2 expression at different time points in breast cancer cells cocultured with adipocytes. Interestingly, 3 h to 72 h of adipocyte coculture increased PLOD2 expression in breast cancer cells, with the most significant upregulation occurring between 48 h and 72 h (Fig. 5c). Finally, we showed that IL-6 (5 ng/ mL) and leptin (50 ng/ mL) significantly increased the migration of MDA-MB-231 and MDA-MB-468 breast cancer cells (Fig. 5d, e). Considering all these observations together, we speculated that the short-term increase in PLOD2 is mainly induced by adipocyte-derived IL-6, while the long-term effects are attributable to both adipocyte-derived leptin and IL-6. Additionally, both of these factors promote breast cancer cell migration.

Our results showed that adipocyte-derived IL-6 and leptin stimulate the migration of tumor cells via upregulation of PLOD2 expression. To confirm the effects of adipocyte-derived IL-6 and leptin on the regulation of distal organ seeding and growth of metastatic tumor cells, we conducted tail vein metastasis assays using the MDA-MB-231 human breast cancer cell line. MDA-MB-231 cells were previously cocultured either with or without adipocytes, cocultured with adipocytes in the presence of a murine IL-6 blocking antibody, or cocultured with adipocytes following OBR depletion. After 3 days, cells were harvested, and resuspended in Cell Tracker™ blue (CMTPX), and intravenously injected into NOD SCID mice. As shown in Fig. 5f, the dissemination of breast cancer cells toward the lungs were enhanced in mice injected with MDA-MB-231 cells previously cocultured with adipocytes compared with mice injected with MDA-MB-231 cells grown alone. Interestingly, when MDA-MB-231 cells were cocultured with adipocytes in the presence of a murine IL-6 blocking antibody, cellular dissemination toward the lungs was abrogated compared with mice injected with the same cells cocultured with adipocytes without the blocking antibody (Fig. 5f). Moreover, depletion of OBR abolished the adipocyte-induced dissemination of MDA-MB-231 cells toward the lungs (Fig. 5f). Histological analysis indicated that both the size and number of metastatic nodules were significantly enhanced in mice injected with adipocyte-cocultured MDA-MB-231 cells compared with MDA-MB-231 cells grown alone (Fig. 5f). Treatment with a murine IL-6 blocking antibody and depletion of OBR significantly decreased both the size and number of the nodules (Fig. 5f).

Therefore, our results clearly suggest that adipocyte-derived IL-6 and leptin promote the invasive phenotype of breast cancer cells via upregulation of PLOD2 both in vitro and in vivo.

### Adipocytes promote PLOD2 expression by activating the JAK/STAT3 and AKT signaling pathways

**Fig. 6:**
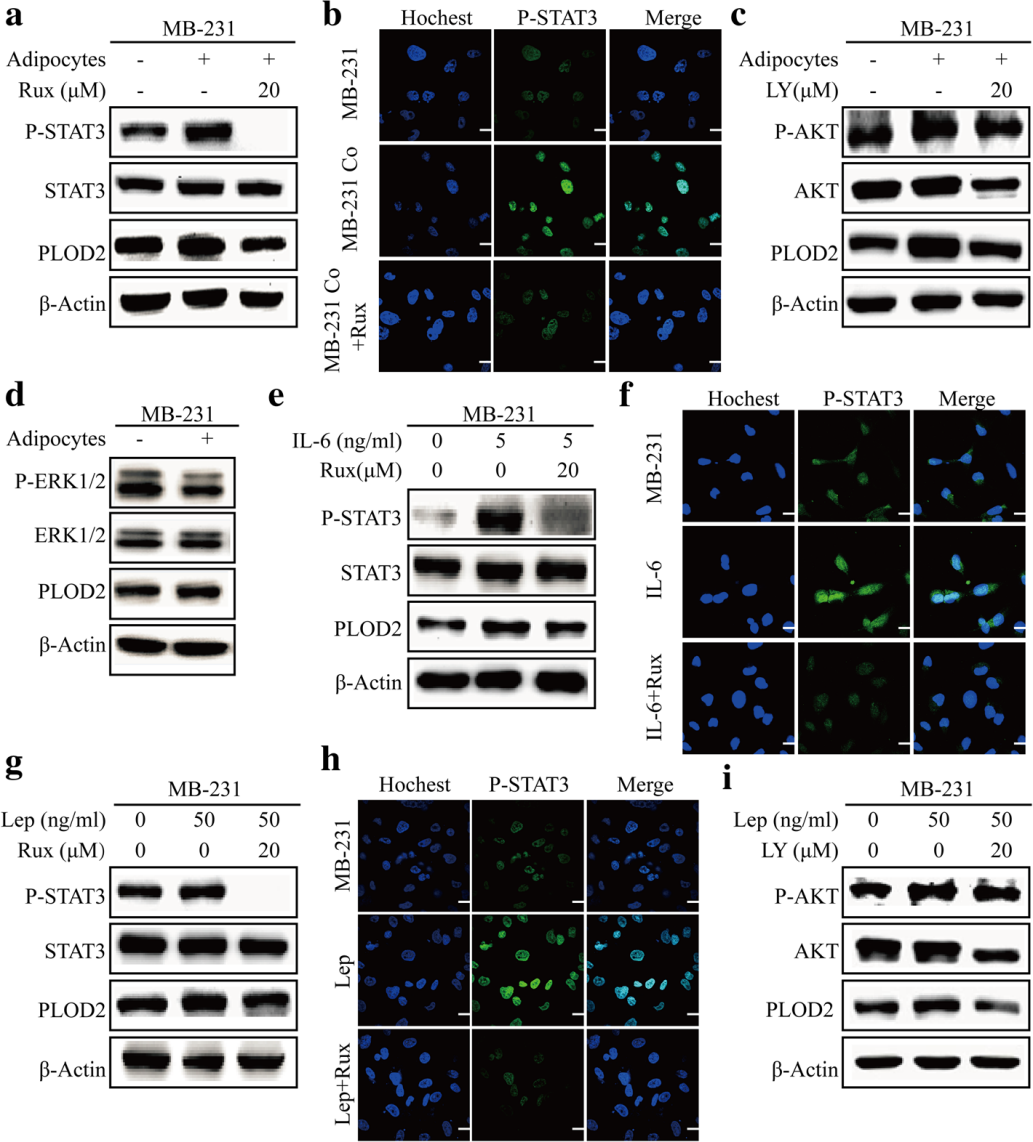
Adipocyte-derived IL-6 and leptin activate the JAK/STAT3 and AKT signaling pathways to promote PLOD2 expression. **a** MDA-MB-231 (MB-231) cells were monocultured or cocultured with adipocytes. An inhibitor directed against janus kinase (ruxolitinib) or PBS was added to the culture medium. After 3 days, tumor cells were harvested to detect protein expression levels of PLOD2, P-STAT3 and STAT3. **b** MDA-MB-231 (MB-231) cells were grown on coverslips in inserts. Cells in the lower chamber were monocultured or cocultured with adipocytes and treated with either ruxolitinib or PBS. After 3 days, cell were fixed and stained for P-STAT3. Nuclei were stained with Hoechst. Scale bars, 20 μm. **c** MDA-MB-231 (MB-231) cells were monocultured or cocultured with adipocytes. Cells were treated with either an inhibitor directed against phosphatidylinositol 3-kinase (LY294002) or PBS. After 3 days, tumor cells were harvested to detect protein expression levels of PLOD2, P-AKT and AKT. **d** MDA-MB-231 (MB-231) cells were monocultured or cocultured with adipocytes. After 3 days, tumor cells were harvested to detect protein expression levels of PLOD2, P-ERK1/2 and ERK1/2. **e** MDA-MB-231 (MB-231) cells were treated with or without IL-6 plus either ruxolitinib or PBS. After 12 h, tumor cells were harvested to detect protein expression levels of PLOD2, P-STAT3 and STAT3. **f** MDA-MB-231 (MB-231) cells were grown on coverslips and treated with or without IL-6. Ruxolitinib or PBS was added to the culture medium. After 12 h, cells were fixed and stained for P-STAT3. Nuclei were stained with Hoechst. Scale bars, 20 μm. **g** MDA-MB-231 (MB-231) cells were treated with or without leptin. Ruxolitinib or PBS was added to the culture medium. After 3 days, tumor cells were harvested to detect protein expression levels of PLOD2, P-STAT3 and STAT3. **h** MDA-MB-231 (MB-231) cells were grown on coverslips and treated with or without leptin. Ruxolitinib or PBS was added to the culture medium. After 3 days, tumors cells were fixed and stained for P-STAT3. Nuclei were stained with Hoechst. Scale bars, 20 μm. **i** MDA-MB-231 (MB-231) cells were treated with or without leptin. LY294002 or PBS was added to the culture medium. After 3 days, tumor cells were harvested to detect protein expression levels of PLOD2, P-AKT and AKT

Previous studies have demonstrated that PLOD2 expression is upregulated by both STAT3 and paracrine signals from cancer-associated fibroblasts (CAFs) [13]. Furthermore, adipocyte-derived adipokines activate cell surface receptors and drive signaling through the Janus kinase (JAK)/signal transducers and activators of transcription (STAT), phosphatidylinositol 3-kinase (PI3K), and mitogen-activated protein kinase (MAPK) signaling pathways, all of which are frequently altered in cancer [7]. Therefore, we postulated that coculture of adipocytes with breast cancer cells promoted PLOD2 expression via activation of the JAK/STAT, MEK/ERK and PI3K/AKT signaling pathways. We tested this hypothesis by coculturing MDA-MB-231 and MDA-MB-468 breast cancer cells with adipocyte. We observed that adipocyte coculture increased PLOD2 expression and activated phosphorylation of STAT3 and AKT (Fig. 6a, c, Additional file 5: Figure S4a, c) but not phosphorylation of ERK (Fig. 6d and Additional file 5: Figure S4d). Inhibition of STAT3 activity using a pharmacological inhibitor of JAK (ruxolitinib) decreased PLOD2 expression in adipocyte cocultured MDA-MB-231 and MDA-MB-468 breast cancer cells (Fig. 6a, Additional file 5: Figure S4a). Additionally, treatment with a phosphatidylinositol 3-kinase inhibitor (LY294002) decreased PLOD2 expression in MDA-MB-231 and MDA-MB-468 breast cancer cells (Fig. 6c, Additional file 5: Figure S4c). Further, three days of coculture in the presence or absence of adipocytes not only increased PLOD2 and P-STAT3 protein levels but also promoted P-STAT3 nuclear accumulation in MDA-MB-231 and MDA-MB-468 cells. Inhibition of the JAK/STAT pathway inhibited P-STAT3 nuclear accumulation (Fig. 6b, Additional file 5: Figure S4b).

Based on the observation that adipocyte-derived IL-6 and leptin promoted PLOD2 expression in breast cancer cells, we next explored whether IL-6 and leptin activated JAK/STAT and AKT signals in vitro. Stimulation with IL-6 significantly increased the expression of PLOD2 and induced STAT3 tyrosine phosphorylation. PLOD2 expression could be inhibited upon treatment with ruxolitinib (Fig. 6e, Additional file 5: Figure S4e). IL-6-stimulated cells also displayed upregulation of STAT3 phosphorylation and a disorganized nuclear state; these phenotypes could be reversed upon treatment with ruxolitinib (Fig. 6f, Additional file 5: Figure S4f). However, AKT signaling was not activated by stimulation with IL-6 (Additional file 5: Figure S4j). A 3-day exposure to leptin significantly increased PLOD2 expression and activated phosphorylation of STAT3 and AKT in MDA-MB-231 and MDA-MB-468 breast cancer cells (Fig. 6 g, i, Additional file 5: Figure S4 g, i). Following treatment with leptin, pharmacological inhibition of the JAK/STAT and AKT signaling pathways with ruxolitinib and LY294002, respectively, decreased PLOD2 expression (Fig. 6g, i, Additional file 5: Figure S4 g, i). Immunofluorescence experiments also confirmed that leptin treatment promoted P-STAT3 nuclear accumulation in MDA-MB-231 and MDA-MB-468 cells and that this nuclear accrual could be inhibited by treatment with ruxolitinib (Fig. 6h, Additional file 5: Figure S4 h). Together, these data reveal that adipocyte-derived IL-6 and leptin activate the JAK/STAT3 and PI3K/AKT signaling pathways to promote PLOD2 expression.

### High PLOD2 expression correlates with poor prognosis of breast cancer and closely relates to GP130 and OBR in clinical samples

**Fig. 7:**
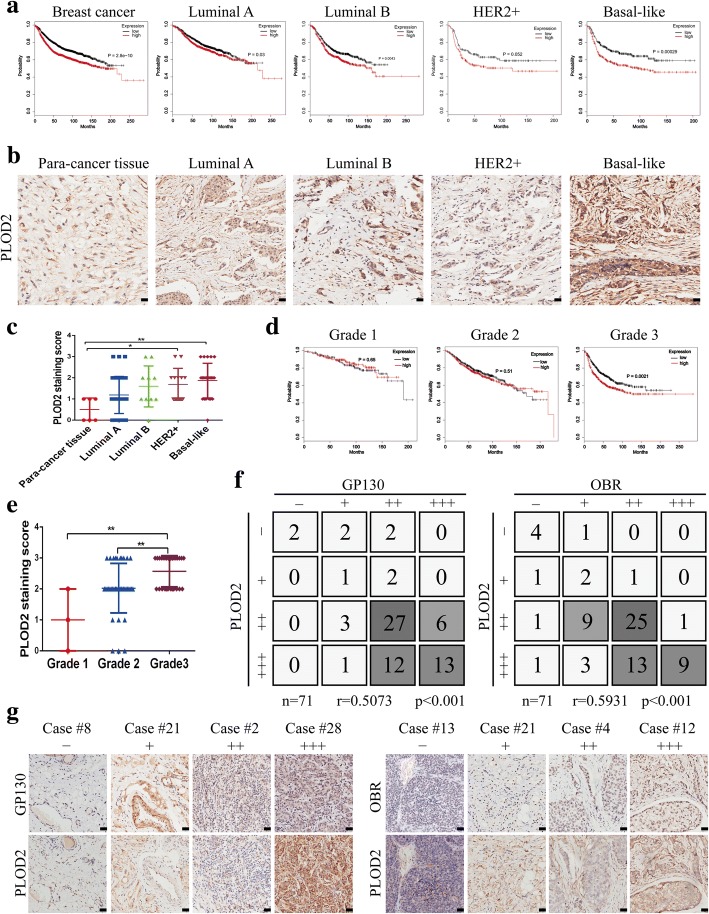
High PLOD2 expression is closely correlated with poor prognosis of breast cancer and closely relates to GP130 and OBR in clinical samples. **a** Kaplan-Meier analysis of relapse-free survival (RFS) in breast cancer patients. PLOD2 in all breast cancer patients (*n* = 1975), in luminal A breast cancer patients (*n* = 966), in luminal B breast cancer patients (*n* = 575), and in basal-like breast cancer patients (*n* = 309). **b** Expression of PLOD2 was analyzed by immunohistochemistry (IHC) in breast cancer specimens of different subtypes and in para-cancerous specimens. Para-cancerous tissue is defined as tissue 2 cm away from the tumor tissue. Scale bars, 50 μm. **c** Scatter plots showing the staining index of PLOD2 in 105 breast cancer specimens. **d** Kaplan-Meier analysis of relapse-free survival (RFS) curve in breast cancer patients with different stages status breast cancer. **e** Scatter plots showing the staining index of PLOD2 in different stages status breast cancer. **f**, **g** Expression level of GP130, OBR and PLOD2 were consistently analyzed in 71 breast cancer specimens (Excluding 4 broken specimens). Scale bars, 50 μm

On the base of the above results, we found that PLOD2 is increased in three human breast cancer cell lines (MDA-MB-231, MDA-MB-468 and SK-BR-3) following coculture with adipocytes. Among them, MDA-MB-231 and MDA-MB-468 were triple-negative breast cancer (TNBC) cells while SK-BR-3 was non-TNBC cells. In order to figure out whether the expression of PLOD2 was distinct from TNBC to non-TNBC. We first analyzed the expression level of PLOD2 using The Cancer Genome Atlas (TCGA) dataset. The results revealed that PLOD2 mRNA expression was much higher in TNBC patients, compared to non-TNBC patients (Additional file 6: Figure S5a). We next examined PLOD2 expression cross a panel of breast cancer cell lines. The results indicated that PLOD2 was much higher expressed in TNBC than that in non-TNBC (Additional file 6: Figure S5b). Next, to explore the potential correlation of PLOD2 with the prognosis of breast cancer, we first analyzed survival correlations using the Kaplan-Meier plotter (http://kmplot.com/analysis/). Our results showed that patients with high PLOD2 expression tended to exhibit a shorter survival time compared to patients with low PLOD2 expression (Fig. 7a). The association of PLOD2 expression with poor prognosis was also significant in different subtypes of breast cancer, especially in basal-like tumors, which exists same characteristics with TNBC (Fig. 7a). These results suggest that PLOD2 is an independent prognostic factor in patients with breast cancer and PLOD2 is much higher expressed in TNBC than that in non-TNBC, but the coculture results show that PLOD2 is upregulated in both TNBC and non-TNBC breast cancer cell lines following coculture with adipocytes, thus, suggesting adipocytes can facilitate pro-metastasis role in TNBC and non-TNBC via PLOD2-dependent way.

The significance of PLOD2 expression in breast cancer was further confirmed by immunohistochemistry in a tumor tissue microarray. The staining index (SI) of PLOD2 was calculated based on staining intensity and the proportion of positive cells, and defined as score 0, 1, 2 and 3. We excluded 35 broken specimens, leaving 105 specimens total in this experiment. We found that PLOD2 was upregulated in breast cancer tissues compared with para-cancerous tissues. We observed significantly increased PLOD2 expression in basal-like tumors, which exists same characteristics with TNBC (Fig. 7b, c). Taken together, these findings reveal that high PLOD2 expression may serve as a clinical biomarker for poor prognosis in breast cancer patients.

Furthermore, we examined the correlations between relapse-free survival (RFS) of different stages status breast cancer and PLOD2 expression were analyzed by Kaplan-Meier plotter. The results showed that the expression of PLOD2 was positively correlated with the stage of breast cancer, and in particular in breast cancer patients at stage III but not at stage I and II (Fig. 7d). The clinical significance of PLOD2 in samples from different stages status was further evaluated by IHC analysis in a tumor tissue microarray. As shown in Fig. 7e, PLOD2 was positively correlated with the stage of breast cancer, respectively, strongly expressed in breast cancer at stage II and III. In addition, above results indicated that adipocyte-derived IL-6 and leptin promote PLOD2 expression through activation of their respective receptors. And further coexpression analysis indicated that GP130 and PLOD2 or OBR and PLOD2 were simultaneously expressed in breast cancer tissue microarray (Fig. 7f, g). Taken together, these results indicated that GP130 and PLOD2, OBR and PLOD2 exhibited a positive correlation. And PLOD2 is positively correlated with the stage of breast cancer.

## Discussion

Several studies have suggested that PLOD2 is dysfunctional in multiple cancer types, including sarcoma, lung cancer, breast cancer, glioblastoma, cervical cancer and bladder cancer [11, 12, 14, 17, 34]. Recent studies have shown that PLOD2 is closely related to cancer metastasis [17]. Mechanistic studies have revealed that HIF-1α, TGF-β, microRNA-26a/b and EGF are involved in the modulation of PLOD2 expression [34, 35]. In addition, paracrine signals from cancer-associated fibroblasts (CAFs) can also upregulate PLOD2 expression in lung cancer [13]. CAFs play a vital role in the tumor microenvironment and are known to secrete several chemokines and cytokines that are involved in cancer progression [36]. Therefore, we speculated that paracrine signals from other stromal cells, such as adipocytes, might also play a role in regulating PLOD2 expression. Adipocytes are a major component of breast tissue and are known to secrete several factors, including adipokines and cytokines, which play a pivotal role in breast cancer progression [37, 38]. Therefore, in this study we investigated whether adipocyte-derived adipokines could promote breast cancer metastasis by regulating PLOD2 expression. The crosstalk between adipocytes and breast cancer cells may have potential clinical significance.

**Fig. 8:**
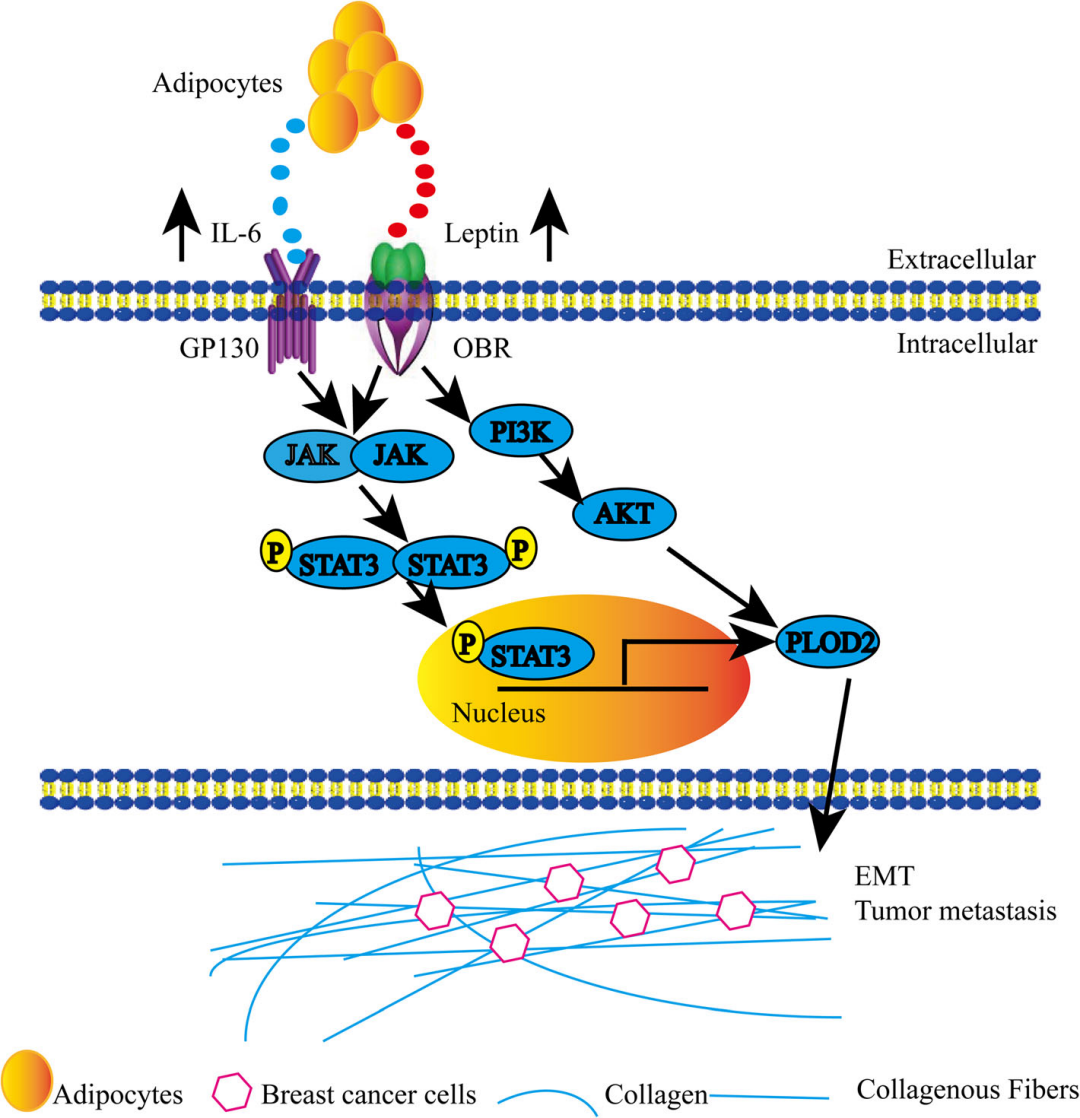
Mechanism of adipocyte-derived IL-6 and leptin-mediated promotion of breast cancer metastasis. Schematic representation of the crosstalk between adipocytes and breast cancer cells. Adipocyte-derived IL-6 and leptin activate GP130 and OBR, which triggers the downstream JAK/STAT3 and PI3K/AKT signaling pathways to promote PLOD2 expression. Ultimately, PLOD2 promotes breast cancer metastasis

Our proposed mechanism demonstrating the crosstalk between adipocytes and breast cancer cells and the role of PLOD2 in mediating adipocyte-driven migration and metastasis is summarized in Fig. 8. Here, we show that PLOD2 is upregulated in breast cancer cells during adipocyte-driven migration and invasion. Further, depletion of PLOD2 attenuates adipocyte-induced breast cancer cell migration in vitro. Moreover, knockdown of PLOD2 inhibits breast cancer cell migration and metastasis both in vitro and in vivo. However, knockdown of PLOD2 had no detectable effect on MDA-MB-231 cell proliferation in colony formation assays, though in vivo experiments revealed that PLOD2-deficient MDA-MB-231 tumors were smaller. Furthermore, increased PLOD2 expression in breast tumors correlated with poor prognosis of breast cancer patients. These findings indicate that PLOD2 directly promotes breast cancer metastasis and mediates adipocyte-driven migration and invasion. In this study, We chose the xenograft model to study the tumor microenvironment interactions which is stable and most commonly used model at present [39, 40]. But actually, Using a xenograft model in this experiment has some disadvantages, for instance, the tumor component is human while the environment is mouse that will lead to insufficiently reflect the physiological environment in humans. Generally, cancer cell line-derived (CDX) or patient-derived (PDX) xenografts in immunodeficient mice have been successfully used in testing of various drugs in preclinical trials, but naturally these models lack the human environment, such as, immune system counterpart, microenvironment and so on [41]. Therefore, in order to solve these problems, we can use the transgenic mouse model of breast cancer to compensate the difference of microenvironment in the xenograft model in our subsequent research, which can verify the role of PLOD2 in breast cancer metastasis.

IL-6 and leptin were identified as important factors involved in cancer progression in a study examining the over-production of inflammatory cytokines due to chronic low-grade inflammation. IL-6 and leptin have been shown to be indispensable for the proliferation, metastasis and initiation of cancer and are significantly associated with poor prognosis in human cancers [42–46]. Our results demonstrated that, of the adipocyte-derived adipokines, IL-6 and leptin were highly expressed after coculture with breast cancer cells. Interestingly, the receptors for IL-6, leptin and PLOD2 were all upregulated upon adipocyte-stimulated migration. Treatment with a murine IL-6 blocking antibody or depletion of OBR abrogated both the expression of PLOD2 and adipocyte-stimulated metastasis, indicating that PLOD2 plays an important role in mediating breast cancer metastasis via adipocyte-derived IL-6 and leptin. Many other adipokines were expressed in this coculture system, in addition to IL-6 and leptin. Thus, we could not exclude the influence of other adipokines on the expression of PLOD2. However, in our study we confirmed that IL-6 and leptin were responsible for the adipocyte-mediated upregulation of PLOD2 in breast cancer. Previous research has suggested that PLOD2 promotes metastasis both directly and also indirectly through the induction of collagen cross-link switch or collagen fiber alignment [12–14]. Further research has suggested that PLOD2 is involved in TGF-β-induced EMT in cervical cancer [14]. In this study, we confirmed that PLOD2 mediates adipocyte-stimulated breast cancer metastasis and promotes the epithelial-mesenchymal transition (EMT) of breast cancer cells, suggesting that PLOD2 directly regulates breast cancer metastasis. However, our study did not confirm whether the adipocyte-induced upregulation of PLOD2 could account for collagen reorganization. Our findings indirectly indicate, however, that adipocyte-derived IL-6 and leptin might influence collagen reorganization, further promoting cancer metastasis, though confirmation of this hypothesis will require further experimentation.

Increasing evidence demonstrates that the JAK/STAT3, PI3K/AKT and MEK/ERK pathways are frequently activated by adipokines, such as IL-6 and leptin and that these pathways are often altered in several types of cancer [42, 47, 48]. Therefore, we explored whether the expression of PLOD2 is regulated by any of these three classical regulatory pathways. In our coculture system, PLOD2 was regulated by the JAK/STAT3 and PI3K/AKT signaling pathways but not by the MEK/ERK signaling pathway. Treatment with IL-6 increased PLOD2 expression by activating the JAK/STAT3 signaling pathway, while stimulation with leptin activated both the JAK/STAT3 and PI3K/AKT signaling pathways to enhance PLOD2 expression. Together, our results suggest that pharmacological inhibition of IL-6 and leptin as well as the downstream JAK/STAT3 and PI3K/AKT signaling pathways can decrease PLOD2 expression and suppress breast cancer metastasis.

Over the past several years, PLOD2 has been implicated as a protumorigenic agent in multiple cancers, with a specifically pivotal role in cancer metastasis. We are the first to report that adipocyte-derived adipokines activate PLOD2 expression via activation of the JAK/STAT3 and PI3K/AKT signaling pathways, thus promoting cancer migration and metastasis. Thus, our research reveals a novel interaction between adipocytes and breast cancer and lays the way for clinical study aimed at exploring the interaction between adipocytes and PLOD2 in obese breast cancer patients. Furthermore, our results may indicate that obese breast cancer patients more likely to show high expression of PLOD2 which predict a poor prognosis. This assumption still need to be confirmed in human model. Further research may provide new insights leading to new therapeutic targets.

## Conclusions

This study provides novel evidence that adipocytes promote breast cancer metastasis via activation of PLOD2. Inhibition of PLOD2 may be sufficient to suppress migration and invasion in vitro and to alleviate lung and liver metastasis of breast cancer cells in vivo. Further investigation revealed that adipocyte-derived IL-6 and leptin promote PLOD2 expression, thus facilitating breast cancer metastasis. PLOD2 upregulation is regulated by the JAK/STAT3 and PI3K/AKT signaling pathways. Therefore, our results suggest that PLOD2 plays a vital role in adipocyte-dependent invasive activity and high PLOD2 expression may predict poor survival in breast cancer patients.

## Additional files

Additional file 1: **Table S1.** Sequences of the primers used to detect gene expression by qRT-PCR. **Table S2.** Characterization of the antibodies used in this study. **Table S3.** Sequences of the small interfering RNAs targeting OBR. (DOCX 15 kb)

Additional file 2: **Figure S1.** Collagen and PLOD2 expression were significantly increased following coculture with adipocytes. a MDA-MB-231 (MB-231) and MDA-MB-468 (MB-468) breast cancer cells were seeded on coverslips, grown for 3 days alone or cocultured with adipocytes, and then fixed and stained for collagen 1. Nuclei were stained with Hoechst. Scale bars, 50 μm. b SK-BR-3 breast cancer cells were cocultured or monocultured with human adipocytes. After 3 days, cells were harvested and PLOD2 protein expression was determined by Western blotting. (TIF 90378 kb)

Additional file 3: **Figuire S2.** Loss of PLOD2 resulted proliferation and metastasis changes both in vitro and in vivo. a The proliferation of PLOD2-knockdown cells was evaluated by colony formation assay. Colonies were stained with crystal violet. b Scatter plots of primary tumor weights. Error bars represent the means ± SD. (*n* = 6/group) ^**^P < 0.01. c Representative immunohistochemical images for PLOD2 expression in tumor tissues. Scale bars, 100 μm. d Representative IHC staining of PLOD2 expression in normal tissues and metastatic modules. Scale bars, 100 μm. (TIF 177911 kb)

Additional file 4: **Figure S3.** Adipocyte-derived IL-6 and leptin regulate PLOD2 expression. a qRT-PCR analysis of the relative expression levels of IGF-BP1, PAI-1, IL-6, MIF, TIMP-1, TIMP-2 and leptin in adipocytes and adipocytes cocultured with MDA-MB-468 (MB-468) breast cancer cells. Error bars represent means ± SD. ^**^P < 0.01. b Dot hybridization analysis of IL-6 and leptin secretion in 3 T3-L1 preadipocytes, adipocytes and adipocytes cocultured with MDA-MB-468 (MB-468) breast cancer cells. c Dot hybridization analysis of IL-6 and leptin secretion in MDA-MB-468 (MB-468) cells and MDA-MB-468 (MB-468) cells cocultured with adipocytes. d MDA-MB-231 (MB-231) and MDA-MB-468 (MB-468) cells were cultured in normal medium (Control) or in CM obtained from 3 T3-L1, adipocytes, or adipocytes previously grown in the presence of tumor cells. After 3 days, cells were collected and PLOD2 protein expression was detected. e SK-BR-3 breast cancer cells were cocultured in the presence or absence of adipocytes. A blocking antibody directed against IL-6 was added to the culture medium of the experimental cells. After 3 days, tumor cells were harvested and PLOD2 protein expression was detected. f OBR was knocked down using two independent siRNAs (siRNA1 and siRNA2) in MDA-MB-231 (MB-231) and MDA-MB-468 (MB-468) cells. qRT-PCR and Western blotting were used to detect OBR expression in negative control and OBR-knockdown cells. Error bars represent means ± SD. ^**^P < 0.01. g PLOD2 expression levels were assessed in MDA-MB-231 (MB-231) and MDA-MB-468 (MB-468) cells after 72 h of IL-6 treatment. h PLOD2 expression levels were assessed in MDA-MB-231 (MB-231) and MDA-MB-468 (MB-468) cells following 0, 3, 6, 12, and 24 h of leptin treatment. (TIF 183822 kb)

Additional file 5: Adipocyte-derived IL-6 and leptin activate the JAK/STAT3 and AKT signaling pathways to promote PLOD2 expression. a MDA-MB-468 cells were monocultured or cocultured with adipocytes. An inhibitor directed against janus kinase or PBS was added to the culture medium. Tumor cells were harvested to detect protein expression. b MDA-MB-468 cells were grown on coverslips in inserts. Cells were cocultured in the presence or absence of adipocytes and either ruxolitinib or PBS was added to the culture medium. Cell were fixed and stained for P-STAT3. c MDA-MB-468 cells were monocultured or cocultured with adipocytes. An inhibitor directed against phosphatidylinositol 3-kinase or PBS was added to the culture medium. Tumor cells were harvested to detect protein expression. d MDA-MB-468 cells were monocultured or cocultured with adipocytes. Tumor cells were harvested to detect protein expression. e MDA-MB-468 cells were treated with or without IL-6. Ruxolitinib or PBS was added to the culture medium. Tumor cells were harvested to detect protein expression. f MDA-MB-468 cells were grown on coverslips and treated with or without IL-6. Ruxolitinib or PBS was added to the culture medium. Cells were fixed and stained for P-STAT3. g MDA-MB-468 cells were treated with or without leptin. Ruxolitinib or PBS was added to the culture medium. Tumor cells were harvested to detect protein expression. h MDA-MB-468 cells were grown on coverslips and treated with or without leptin. Ruxolitinib or PBS was added to the culture medium. Tumors cells were fixed and stained for P-STAT3. i MDA-MB-468 cells were treated with or without leptin. LY294002 or PBS was added to the culture medium. Tumor cells were harvested to detect protein expression. j MDA-MB-468 cells were treated with or without IL-6 for different times. Cells were harvested to detect protein expression. (TIF 244545 kb)

Additional file 6: **Figure S5.** PLOD2 is markedly upregulated in triple negative breast cancer. a PLOD2 mRNA levels in breast cancer tissues were assessed in 625 non-TNBC and 79 TNBC tissues. Data were acquired from the TCGA database. b Real-time PCR analysis (up) and Western blotting analysis (down) determining the expression of PLOD2 in several breast cancer cell lines. (TIF 101457 kb)

## Abbreviations

3D: Three-dimensional
CAA: Cancer-associated adipocyte
CAFs: Cancer associated fibroblasts
CM: Conditioned medium
ECL: Enhanced Chemiluminescence
ECM: Extracellular matrix
EMT: Epithelial-to-mesenchymal transition
ETP: Endotrophin
F-actin: Filamentous actin
H: Hours
IGF-1: Insulin-like growth factor 1
IL-6: interleukin-6
LOX: Lysyl oxidase
MCP-1: Monocyte chemotactic protein-1
Min: Minutes
OBR/LEPR: leptin receptor
P4HA1: Prolyl 4-hydroxylase subunit alpha-1
PAI-1: Plasminogen activator inhibitor 1
PLOD2: Lysyl hydroxylase 2
qRT-PCR: Quantitative real time PCR
SI: Staining index
TIMP1: Tissue inhibitor of metalloproteinase 1
TNF-α: Tumor necrosis factor
ZO-1: Zona occludens
