# Supplementary material for: Adipocyte-derived IL-6 and leptin promote breast Cancer metastasis via upregulation of Lysyl Hydroxylase-2 expression
Source: Cell Commun Signal. 2018 Dec 18;16:100. doi: 10.1186/s12964-018-0309-z (PMC6299564; doi:10.1186/s12964-018-0309-z)
Supplement: Supplementary file 1 — Table S1. Sequences of the primers used to detect gene expression by qRT-PCR. Table S2. Characterization of the antibodies used in this study. Table S3. Sequences of the small interfering RNAs targeting OBR. (DOCX 15 kb) [file 12964_2018_309_MOESM1_ESM.docx]

Supplementary Table 1: Sequences of the primers used to detect genes expression by qRT-PCR

| Name | Forward | Reverse |
| --- | --- | --- |
| H-18s | GAGGGAGCCTGAGAAACGG | GTCGGGAGTGGGTAATTTGC |
| H-PLOD1 | GCCGTTTGTGTCCCTGTTCTTC | ATGCTGTGCCAGGAACTCTTCC |
| H-PLOD2 | GACAGCGTTCTCTTCGTCCTCA | CTCCAGCCTTTCGTGGTGACT |
| H-PLOD3 | CGAGTGTGAGTTCTACTTCAGCC | CCAGAAGTTGGACCACAGCTTG |
| H-P4HA1 | GCCAAAGCTCTGTTACGTCTCC | CAAAGCAGTCCTCAGCCGTTAG |
| H-LOX | GATACGGCACTGGCTACTTCCA | GCCAGACAGTTTTCCTCCGCC |
| H-OBR | GCAGTCTATGCTGTTCAGGTGC | CCAAAATTCAGGTCCTCTCATAGC |
| H-GP130 | CACCCTGTATCACAGACTGGCA | TTCAGGGCTTCCTGGTCCATCA |
| M-GAPDH | CATCACTGCCACCCAGAAGACTG | ATGCCAGTGAGCTTCCCGTTCAG |
| M-Leptin | GCAGTGCCTATCCAGAAAGTCC | GGAATGAAGTCCAAGCCAGTGAC |
| M-IL-6 | TACCACTTCACAAGTCGGAGGC | CTGCAAGTGCATCATCGTTGTTC |
| M-PAI-1 | CTCATCAGCCACTGGAAAGGCA | GACTCGTGAAGTCAGCCTGAAAC |
| M-IGF-BP1 | GCCCAACAGAAAGCAGGAGATG | GTAGACACACCAGCAGAGTCCA |
| M-MIF | GAACCGCAACTACAGTAAGCTCG | ACGTTGGCAGCGTTCATGTCGT |
| M-TIMP-1 | TCTTGGTTCCCTGGCGTACTCT | GTGAGTGTCACTCTCCAGTTTGC |
| M-TIMP-2 | AGCCAAAGCAGTGAGCGAGAAG | GCCGTGTAGATAAACTCGATGTC |

Supplementary Table 2: Characterization of the antibodies used in the study

| Target | Type | Supplier |
| --- | --- | --- |
| Actin | Monoclonal | Proteintech |
| GAPDH | Polyclonal | ABclonal |
| PLOD2  IL-6  Leptin | Polyclonal  Polyclonal  Polyclonal | ABclonal  ABclonal  ABclonal |
| GP130 | Polyclonal | Wanleibio |
| LEPR(OBR) | Polyclonal | Abcam |
| P-STAT3 | Polyclonal | Cell Signaling Technology |
| STAT3 | Polyclonal | Cell Signaling Technology |
| P-AKT | Polyclonal | Cell Signaling Technology |
| AKT | Polyclonal | Cell Signaling Technology |
| P-ERK1/2 | Polyclonal | Cell Signaling Technology |
| ERK1/1 | Polyclonal | Cell Signaling Technology |
| Collagen 1 | Polyclonal | Cell Signaling Technology |

Supplementary Table 3: Sequences of the small interfering RNA for target OBR

| Name | Sequences |
| --- | --- |
| Negative Control | UUCUCCGAACGUGUCACGUTT  ACGUGACACGUUCGGAGAATT |
| LEPR-1614 | CCAUCUAUUCAUCCCAUAUTT  AUAUGGGAUGAAUAGAUGGTT |
| LEPR-2830 | CCUGGGCACAAGGACUUAATT  UUAAGUCCUUGUGCCCAGGTT |
| LEPR336 | GCUGGACUCUCAAAGAAUATT  UAUUCUUUGAGAGUCCAGCTT |
